# Supplementary material for: ASIC1a affects hypothalamic signaling and regulates the daily rhythm of body temperature in mice
Source: Commun Biol. 2023 Aug 17;6:857. doi: 10.1038/s42003-023-05221-2 (PMC10435469; doi:10.1038/s42003-023-05221-2)
Supplement: Supplementary file 2 — Supplementary information [file 42003_2023_5221_MOESM2_ESM.pdf]

Supplementary Information

ASIC1a affects hypothalamic signaling and regulates the daily rhythm of body temperature in mice

Zhong Peng, Panos G. Ziros, Tomaz Martini, Xiao-Hui Liao, Ron Stoop, Samuel Refetoff, Urs Albrecht, Gerasimos P. Sykiotis, and Stephan Kellenberger

Correspondence: Stephan Kellenberger (stephan.kellenberger@unil.ch)

This PDF file includes:

- Supplementary Figures 1 to 6
- Supplementary Table 1

An additional supplementary information file in this study includes the following:

- Supplementary Data 1: Raw data of graphs and charts

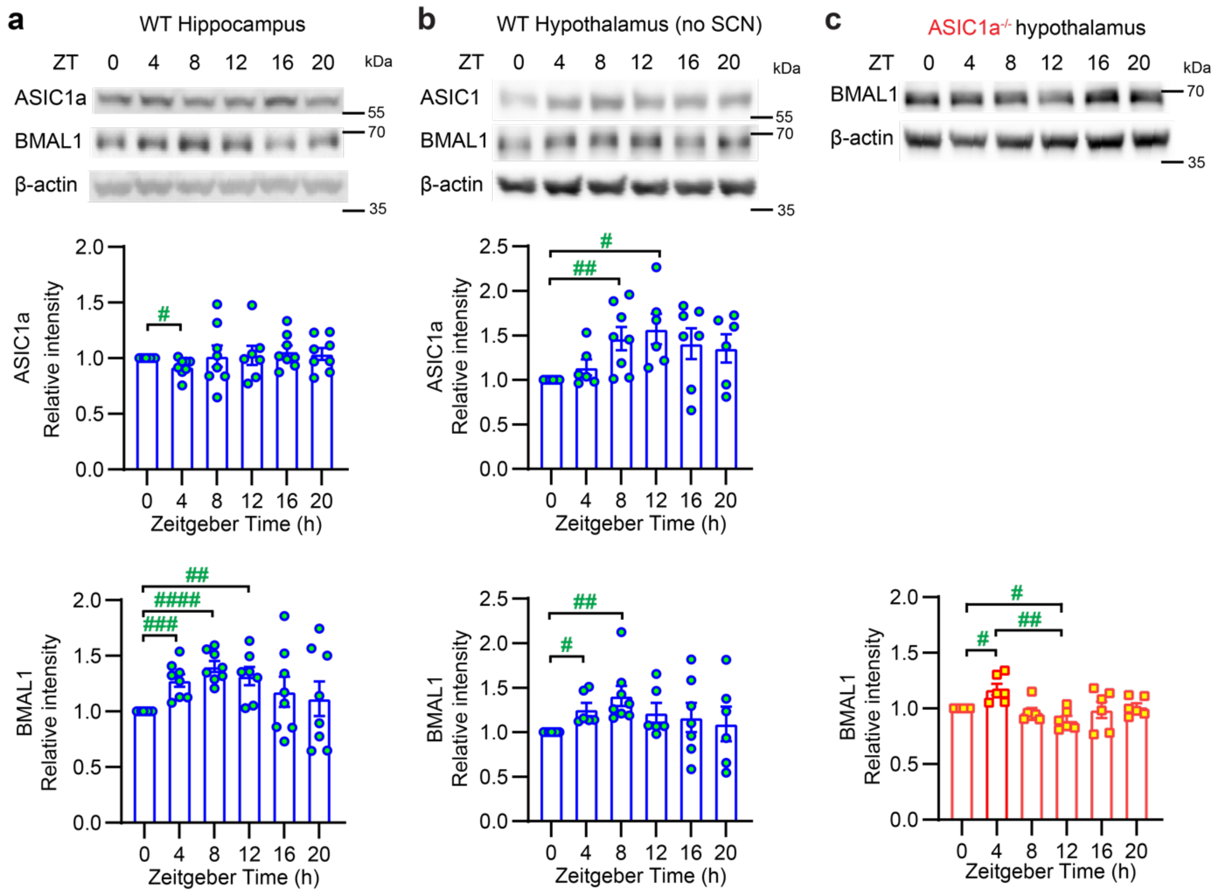

Supplementary Figure 1. Daily expression pattern of ASIC1a and BMAL1 in the hippocampus and in the hypothalamus without SCN. Representative Western blots and quantitative analysis of ASIC1a, BMAL1 and β-actin expression at the indicated ZT in WT hippocampus (**a**), WT hypothalamus without SCN (**b**) and of BMAL1 in ASIC1a$^{-/-}$ hypothalamus (**c**). β-actin was used as a loading control. For the quantification of ASIC1a and BMAL1 expression, the intensity of each protein band is normalized to the corresponding band at ZT0 in each independent experiment, *n*=6-8 animals per condition. $^{\#}$, p<0.05; $^{\#\#}$, p<0.01; $^{\#\#\#}$, p<0.001; $^{\#\#\#\#}$, p<0.0001 compared with each other by one-way ANOVA test and Dunnett's *post-hoc* test or with the ZT0 condition by one-sample t-test, done with log2-transformed data. Error bars indicate SEM.

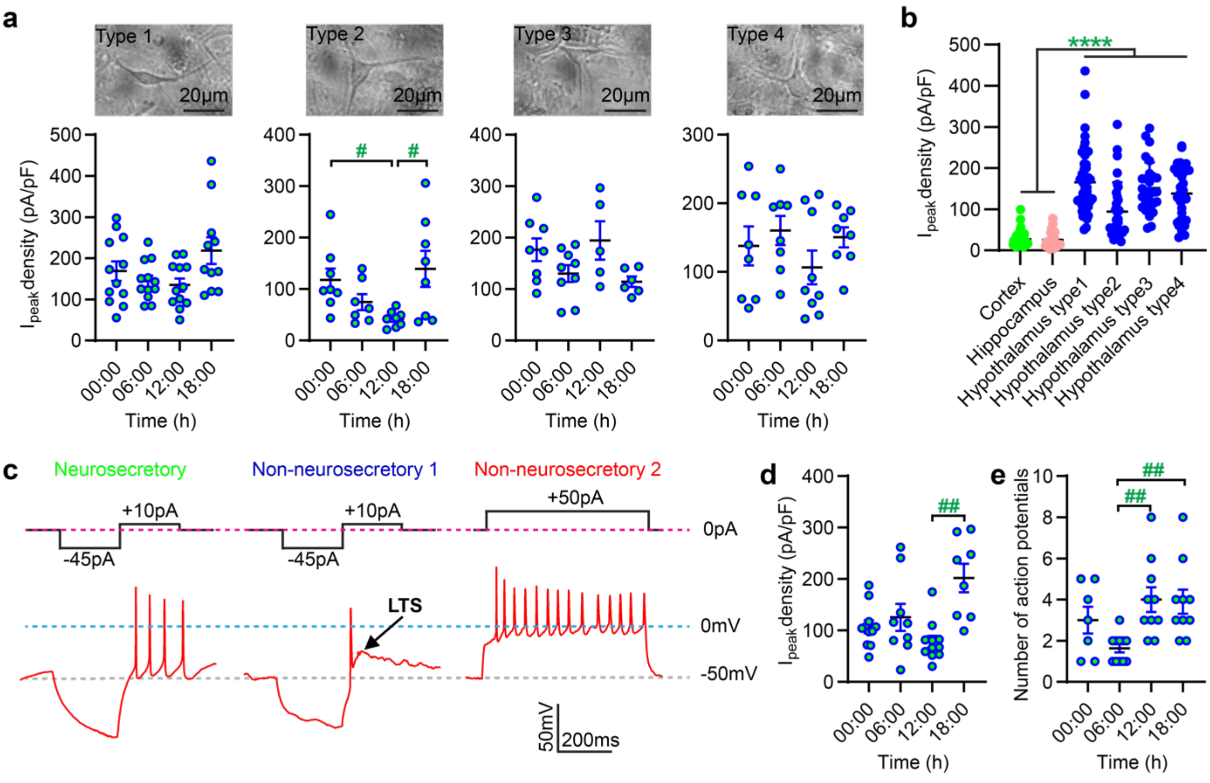

Supplementary Figure 2. Daily rhythm of ASIC currents in cultured hypothalamus neurons. ASIC activity from cultured hypothalamus neurons or as indicated, measured as current density by whole-cell patch-clamp at -60 mV (**a, b, d**) or measured as change in membrane potential (**c**) or number of action potentials (APs, **e**) from current-clamp experiments. Note that these neurons were not synchronized, and the time is indicated as time, not *Zeitgeber* time. **a** Representative images of four typical morphology-based types of cultured hypothalamus neurons at day 12, and peak current densities of pH6.6-induced current at the indicated time are indicated for each of the four types of hypothalamus neurons, *n*=5-12 cells per condition. **b** Peak current densities of pH6.6-induced current in cultured mouse brain neurons, as indicated, over one diurnal cycle, *n*=26-43 cells per condition. **c** Electrogenic properties of cultured hypothalamus neurons to classify them into neurosecretory and non-neurosecretory neurons [15, 16]. The protocols shown in the left and center panels were applied first. The neuron responded with APs only (neurosecretory) or with low-threshold spike (LTS, non-neurosecretory 1). If these two protocols did not induce APs, the current protocol shown on the right was applied. If a burst of APs was induced, the neuron was classified as "non-neurosecretory 2", and if not as "others", as reported in Fig. 2a. **d-e** Data from cultured mouse neurosecretory type 1 hypothalamus neurons. **d** Peak current densities of pH6.6-induced current at the indicated time, *n*=8-11 cells. **e** Number of pH6.6-induced APs at the indicated time, *n*=7-11 cells. Error bars indicate SEM. For statistical analyses of current densities (**a**, **b**,

d), log2-transformed data were compared by one-way ANOVA test and Dunnett's *post-hoc* test. In **e**, non-transformed data were compared with Kruskal-Wallis and Dunn's post-hoc test; $^{\#}$, $p<0.05$; $^{\#\#}$, $p<0.01$; $^{\#\#\#\#}$, $p<0.0001$.

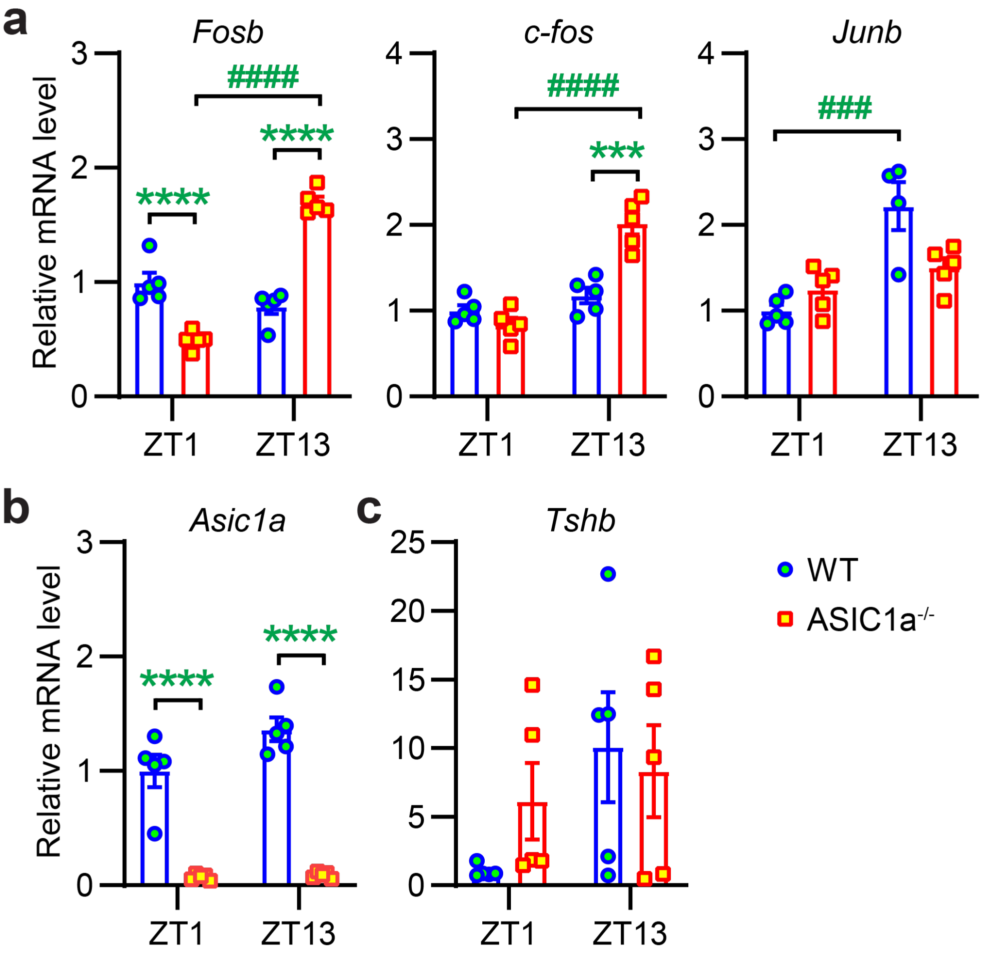

Supplementary Figure 3. Daily expression pattern of hypothalamic genes. **a-d** RT-qPCR analysis to identify the gene expression change of early response genes (**a**), *Asic1a* (**b**), and *thyroid-stimulating hormone β subunit (Tshb,* **c**). Results for each mouse are presented as relative expression normalized to the mean of the WT at ZT1 group. Data are presented as mean ± SEM, *n*=4-5 animals per condition. ***, $p<0.001$; ****, $p<0.0001$; compared WT to corresponding ZT ASIC1a$^{-/-}$; $^{\#\#\#}$, $p<0.001$; $^{\#\#\#\#}$, $p<0.0001$; comparison of each ZT with the corresponding genotype; two-way ANOVA test and Holm-Sidak's *post-hoc* test. Statistical analysis was done from log2-transformed data.

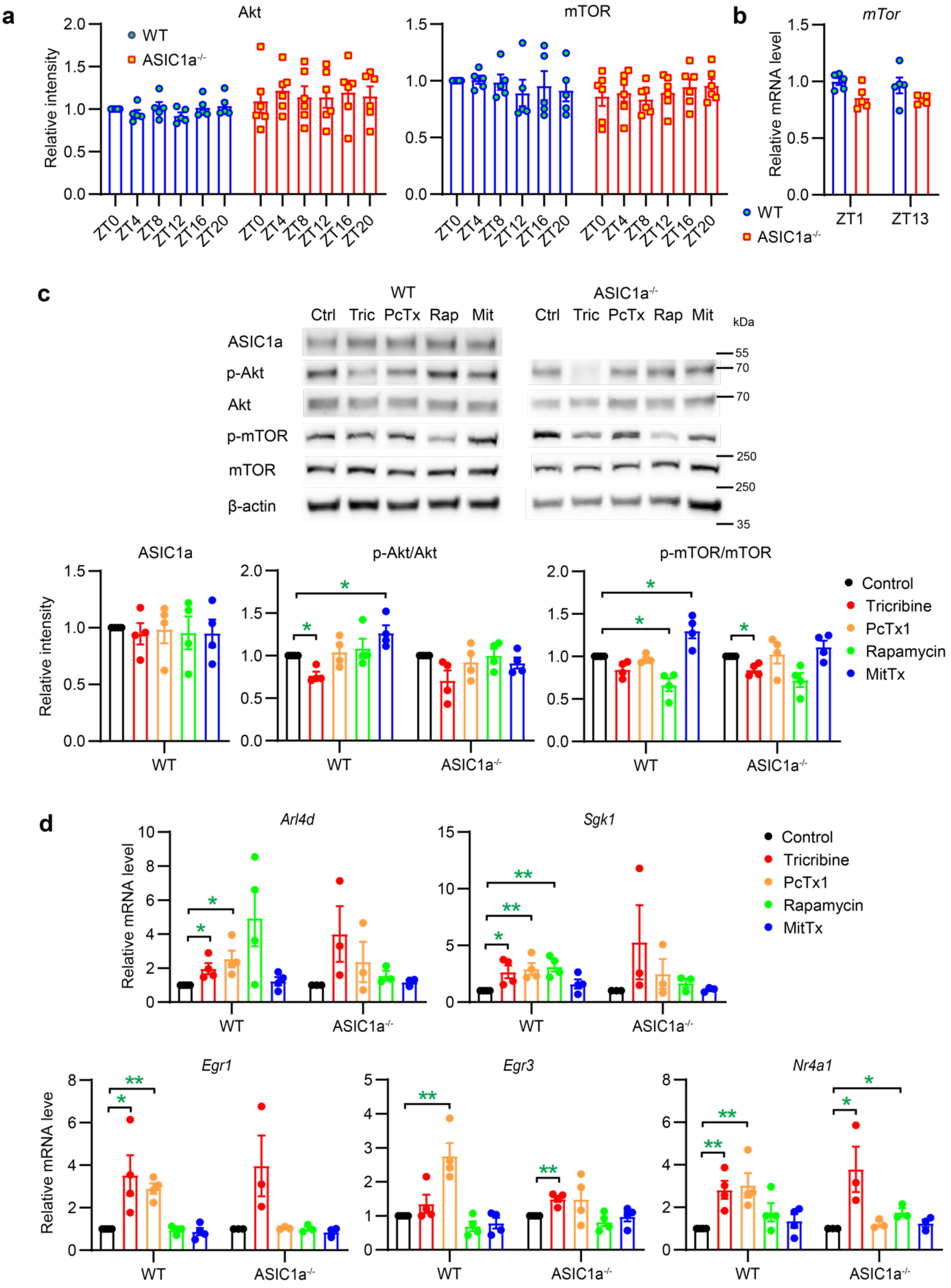

Supplementary Figure 4. Regulation of the Akt-mTOR pathway. a Quantification of Akt and mTOR expression from the independent experiments of hypothalamus tissue shown in Fig. 5a. β-actin was used as a control for the total protein. The intensity of each protein band is

normalized to the corresponding WT band at ZT0 in each independent experiment; *n*=5-6 animals per condition. **b** RT-qPCR analysis of *mTor* mRNA. Results for each mouse are presented as relative expression normalized to the mean of the WT at ZT1; *n*=5 animals per condition. **c** Representative Western blots and quantitative analysis of ASIC1a, Akt, p-Akt, mTOR, p-mTOR and β-actin expression are shown for each protein in WT and ASIC1a$^{-/-}$ cultured cortical neurons treated with triciribine (10µM), PcTx1 (10nM), rapamycin (200nM), MitTx (2nM) or vehicle (control) for 2 h, as indicated. For the quantification of ASIC1a expression, p-Akt/Akt ratio and p-mTOR/mTOR ratio, the values measured for each condition are normalized to the corresponding control value in each independent experiment; *n*=4 independent experiments. *, $p<0.05$; **, $p<0.01$, relative to the control condition in the respective genotype. For p-Akt/Akt in ASIC1a$^{-/-}$, triciribine vs. control, p=0.125; for p-mTOR/mTOR, triciribine vs control in WT, p=0.056; rapamycin vs. control in ASIC1a$^{-/-}$, p=0.066. **d** RT-qPCR analysis of *Arl4d, Sgk1, Egr1, Egr3* and *Nr4a1* expression in cultured hypothalamus neurons (from the same samples as those used in **Fig. 5b-d**). Cultured neurons were treated with triciribine, PcTx1, rapamycin, MitTx or vehicle (control) for 2 h, as indicated. Expression levels measured for each condition are presented relative to the mean of the control in the respective experiment; *n*=3-4 independent experiments. *Egr1*, Triciribine vs. control in ASIC1a$^{-/-}$, p=0.07. (**c** and **d**) *, $p<0.05$; **, $p<0.01$; compared with control by one sample t-test. Statistical analysis was done from log2-transformed data. Data are presented as mean ± SEM.

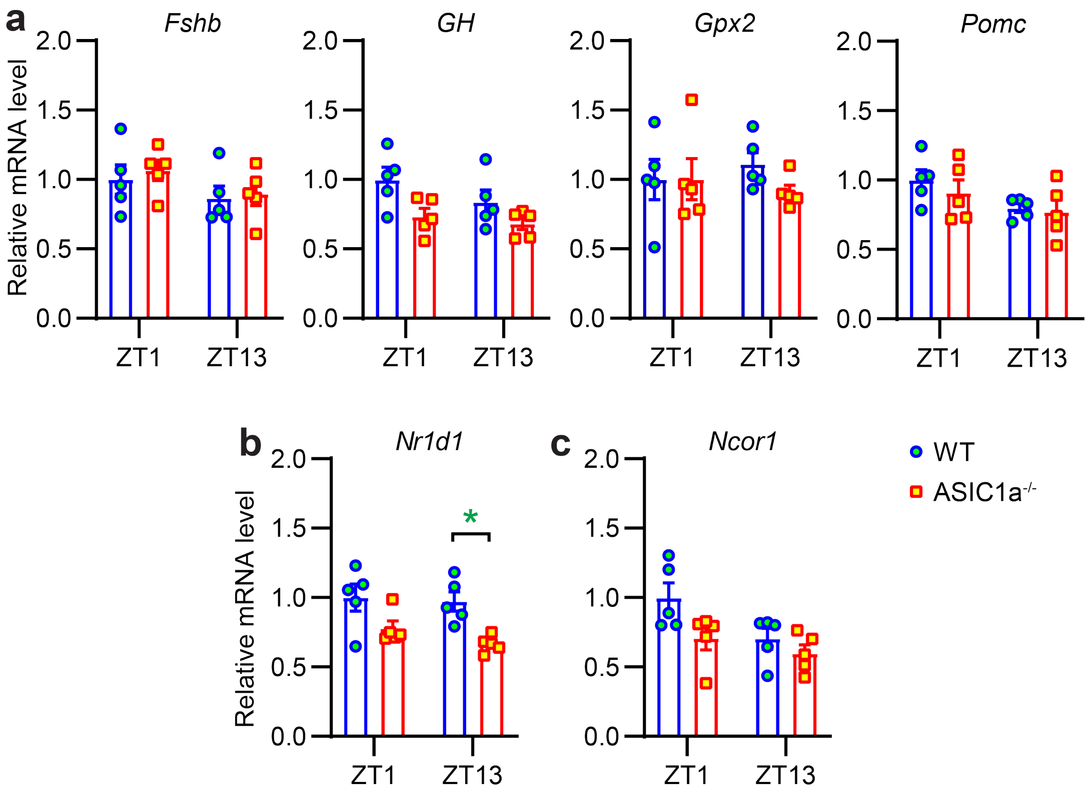

Supplementary Figure 5. Daily expression pattern of molecules and regulators of the HPT axis. mRNA levels in the *pars distalis* (PD) of the pituitary were quantified by RT-qPCR. **a** Follicle-stimulating hormone beta subunit (*Fshb*), growth hormone (*GH*), glutathione peroxidase 2 (*Gpx2*), pro-opiomelanocortin (*Pomc*). **b** Nr1d1 (Rev-Erbα); **c** Ncor1 (Nuclear corepressor 1). Results for each mouse are presented as relative expression normalized to the mean of the WT at ZT1. Data are presented as mean ± SEM, *n*=4-5 animals per condition. *, $p<0.05$; compared between WT and ASIC1a$^{-/-}$ at the same ZT; two-way ANOVA test and Holm-Sidak's *post-hoc* test. Statistical analysis was done from log2-transformed data.

Supplementary Fig. 6 Uncropped blot images

(Figure 1a)

ASIC1a

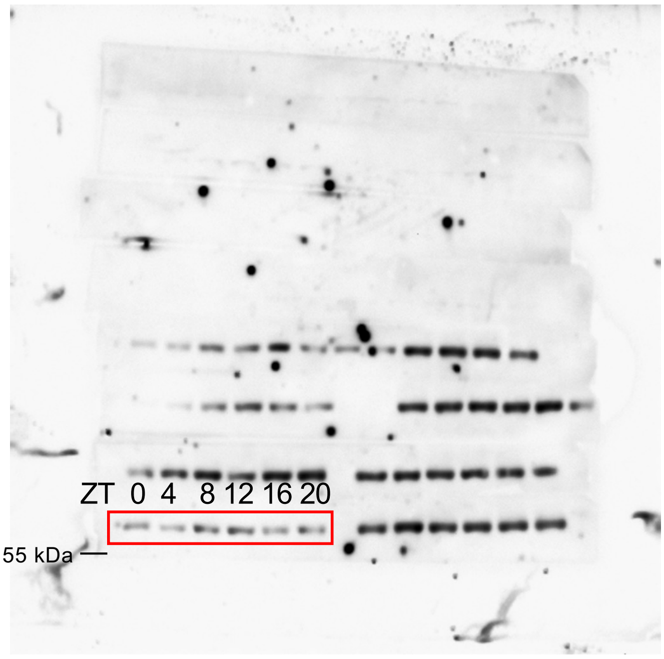

BMAL1

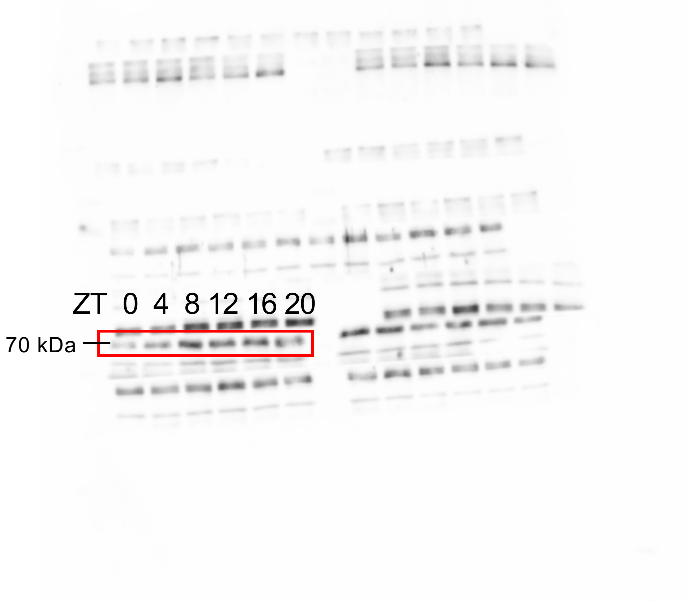

β-actin

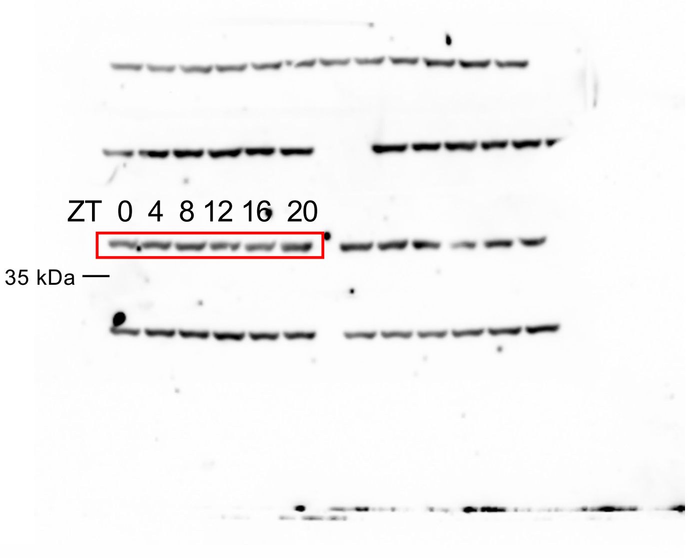

BMAL1

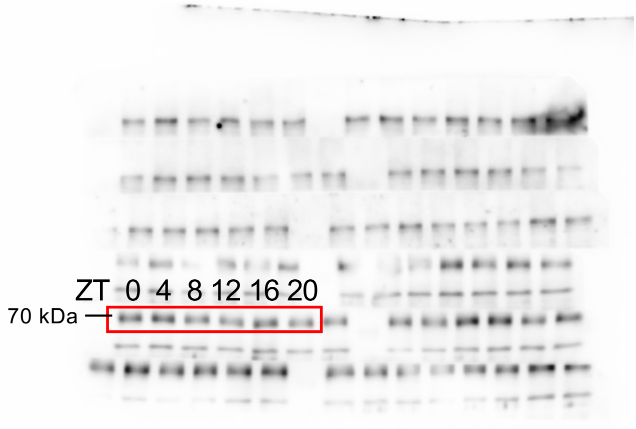

(Figure 1b)

ASIC1a

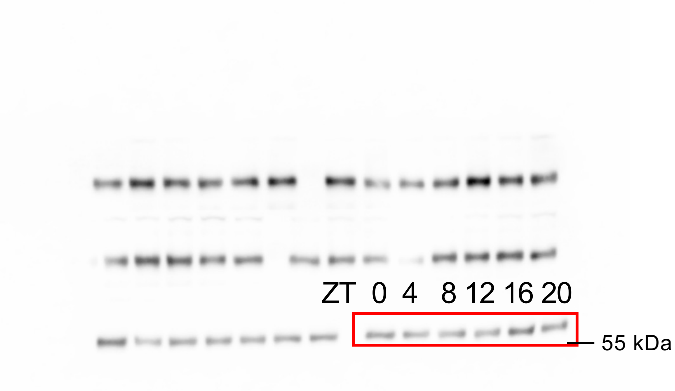

β-actin

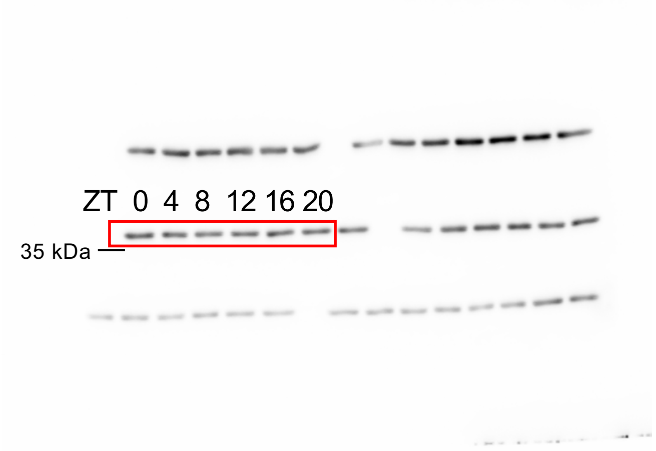

Supplementary Fig. 6, continued

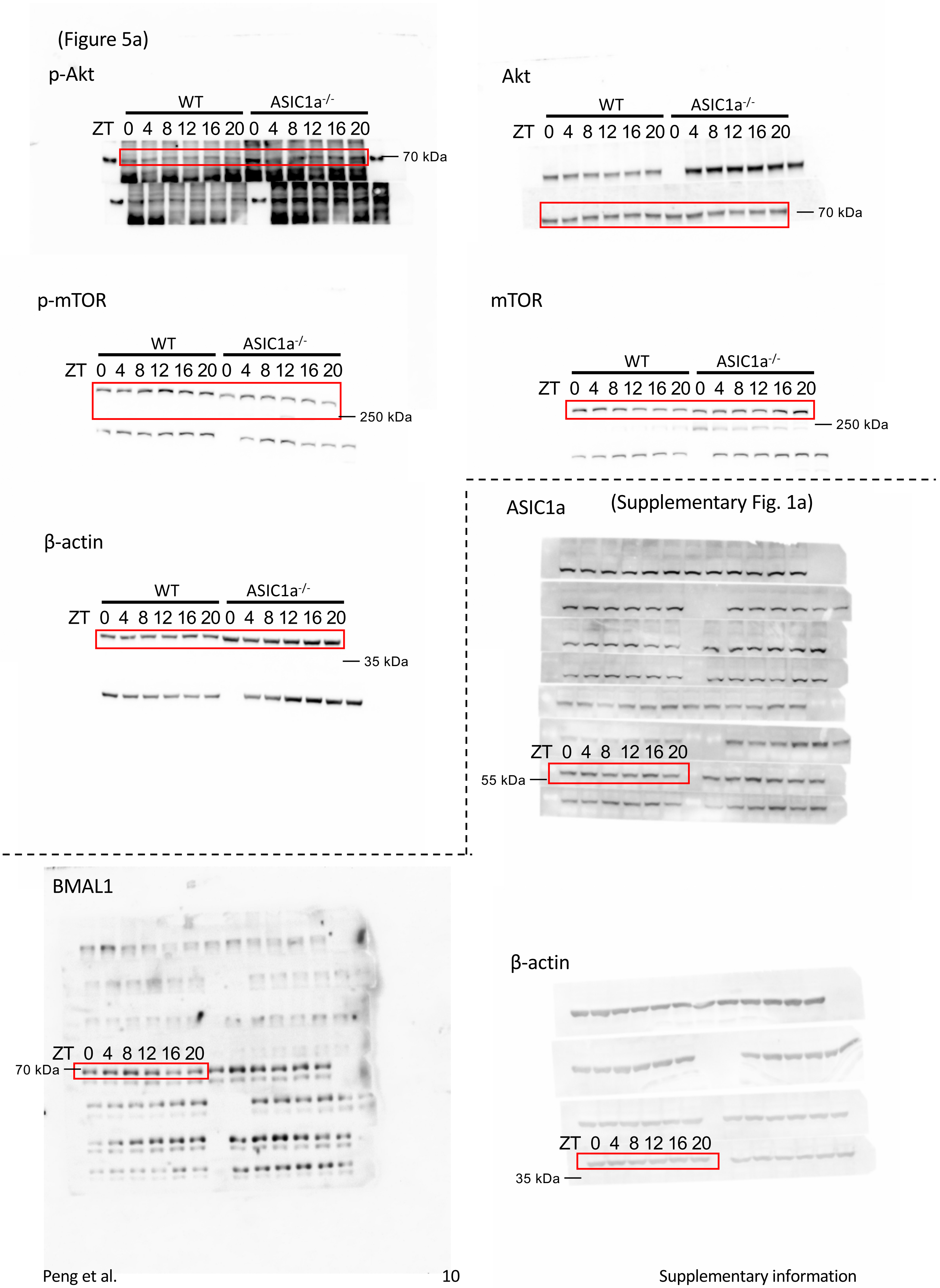

(Supplementary Figure 1b)

ASIC1a

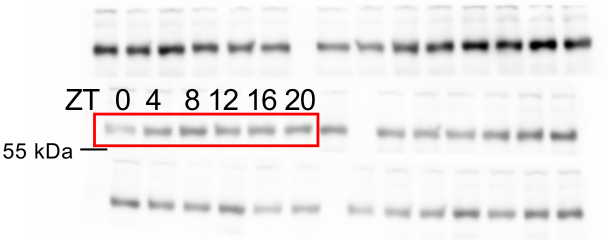

BMAL1

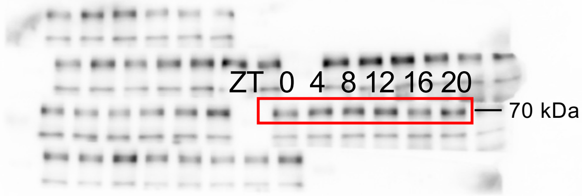

β-actin

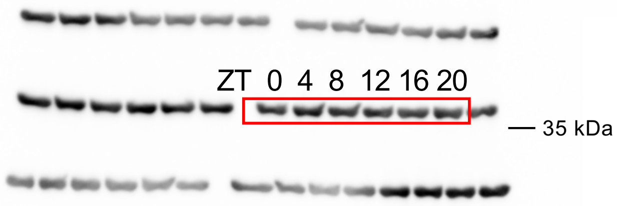

(Supplementary Figure 1c)

BMAL1

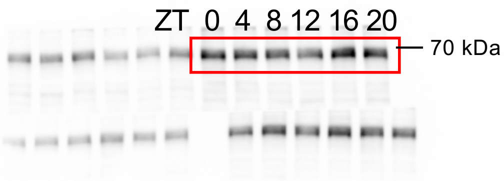

β-actin

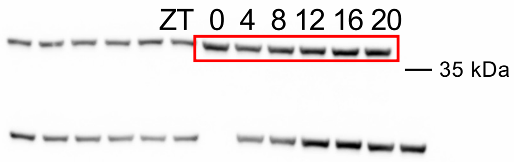

Supplementary Fig. 6, continued

(Supplementary Figure 4c)

ASIC1a (WT)

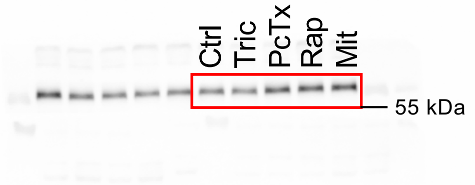

p-Akt (WT)

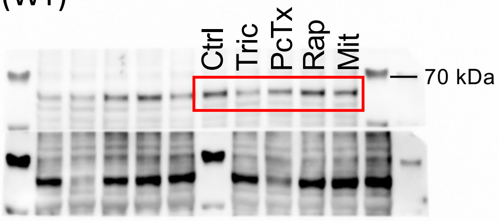

p-Akt (ASIC1a$^{-/-}$)

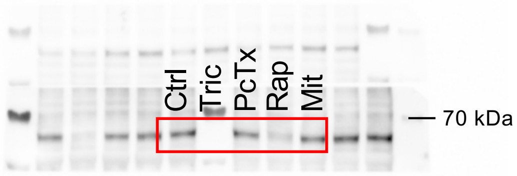

Akt (WT)

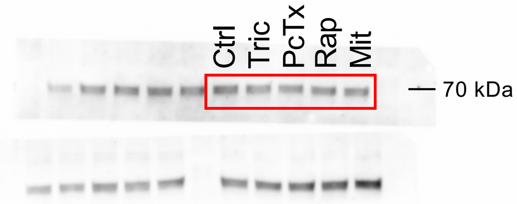

Akt (ASIC1a$^{-/-}$)

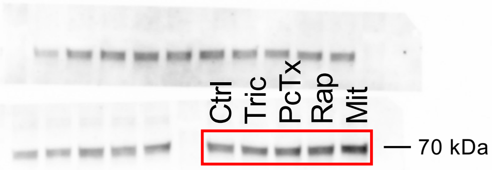

(Supplementary Figure 4c, continued)

p-mTOR (WT)

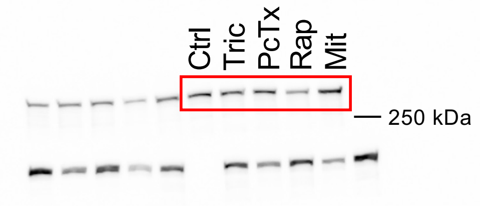

p-mTOR (ASIC1a$^{-/-}$)

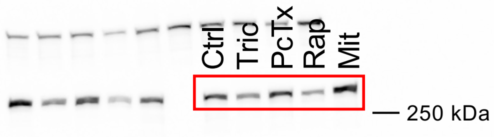

mTOR (WT)

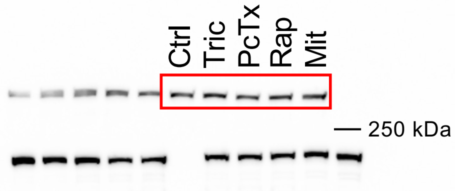

mTOR (ASIC1a$^{-/-}$)

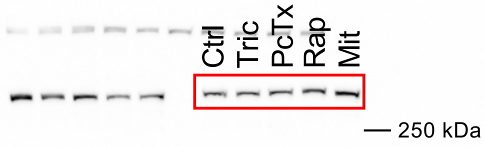

β-actin (WT)

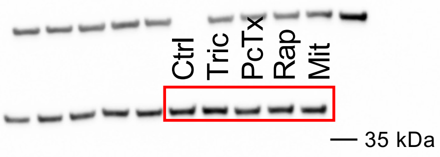

β-actin (ASIC1a$^{-/-}$)

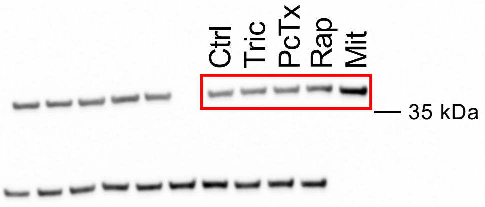

Supplementary Table 1, Primers for RT-qPCR experiments

Gene	Forward primer	Reverse primer
Fshb	AGGGAGGAAAGGAAAGTGGA	AGCCAGCTTCATCAGCATTT
Gh	ACGCGCTGCTCAAAAACTAT	GCTAGAAGGCACAGCTGCTT
Gpx2	GTGCTGATTGAGAATGTGGC	AGGATGCTCGTTCTGCCCA
Prl	CTCAGGCCATCTTGGAGAAG	TCGGAGAGAAGTCTGGCAGT
Tshb	TCAACACCACCATCTGTGCT	TTGCCACACTTGCAGCTTAC
Asic1a	CCTGCTCAACAACAGGTATG	CTCGTCCTGACTGTGGATCT
Gapdh	AACGGGAAGCCCATCACC	CATACTCAGCACCGGCCTCA
Bmal1	TGACCCTCATGGAAGGTTAGAA	GGACATTGCATTGCATGTTGG
Ppia	AGCACTGGGGAGAAAGGATT	CATGCCTTCTTTCACCTTCC
Trh	TCCTGGATCACAAAACGCCA	CTTGTCTTGGTTGGCACGTC
Cers5	GACTGCTTCCAAAGCCTTGAG	GCAGTTGGCACCATTGCTAG
Sgk1	GGG TGC CAA GGA TGA CTT TA	CTC GGT AAA CTC GGG ATC AA
Ddit4	CAAGGCAAGAGCTGCCATAG	CCGGTACTTAGCGTCAGGG
Btg2	CCCCCCGGTGGCTGCCTCCTATG	GGGTCGGGTGGCTCCTATCTA
Nr4a1	TCTGGTCCTCATCACTGATCGA	AATGCGATTCTGCAGCTCTTC
Nr4a3	CAGTGTCGGGATGGTTAAGGAA	CAGACGACCTCTCCTCCCTTT
Arl4d	GCCTCGAGGGCTGAAGACACCCCAGCTT	CTGAATTCGCCTTGCTGATCCGGTGTAA
Egr1	GAACAACCCTATGAGCACCTGAC	CGAGTCGTTTGGCTGGGATA
Egr2	TCAATGTCACTGCCGCTGAT	AGAAATGATCTCTGCAACCAGAA
Egr3	GATCCACCTCAAGCAAAAGG	CGGTGTGAAAGGGTGGAAAT
Hprt	CAGTCCCAGCGTCGTGATTA	TGGCCTCCCATCTCCTTCAT
TrhR	CTTCTTAAACCCCATTCCTT	TTCCTGGAAGATACAGTGCT
c-fos	CGAAGGGAACGGAATAAGATG	GCTGCCAAAATAAACTCCAG
Fosb	ACAGATCGACTTCAGGCGGA	GTTTGTGGGCCACCAGGAC
Junb	ATCCTGCTGGGAGCGGGGAACTGAGGGAAG	AGAGTCGTCGTGATAGAAAGGC

mTor	ATT CAA TCC ATA GCC CCG TC	TGC ATC ACT CGT TCA TCC TG
Pomc	CATAGATGTGTGGAGCTGGTG	CATCTCCGTTGCCAGGAAACAC
TshR	ATCGCGGATCCGAAGTAGCCCAGAGGGTCCCTTGG	GATCAGAATTCCAAGGCTGTTTGCTTATACTCTTC
Ncor1	GAAGCCACAGCAGAAGAACC	ACGACCATGTTCTACCAGGC
Nr1d1	CTTCATCCTCCTCCTCCTTCTA	GTAATGTTGCTTGTGCCCTTG
Dio2	CTTCCTCCTAGATGCCTACAAAC	GGCATAATTGTTACCTGATTCAGG
Dio3	CCGCTCTCTGCTGCTTCAC	CGGATGCACAAGAAATCTAAAAGC
Hprt	CAGTCCCAGCGTCGTGATTA	TGGCCTCCCATCTCCTTCAT

The sequences of the primers are provided.
