# Supplementary material for: ASIC1a affects hypothalamic signaling and regulates the daily rhythm of body temperature in mice
Source: Commun Biol. 2023 Aug 17;6:857. doi: 10.1038/s42003-023-05221-2 (PMC10435469; doi:10.1038/s42003-023-05221-2)
Supplement: Supplementary file 3 — Description of Additional Supplementary Files [file 42003_2023_5221_MOESM3_ESM.pdf]

Description of Additional Supplementary Files

File name: Supplementary Data 1

Description: All source data behind the graphs in the paper.
